# The role of faith-based organizations in the pharmaceutical systems of low-and-middle-income countries – A scoping review

**DOI:** 10.1371/journal.pgph.0006835

**Published:** 2026-07-27

**Authors:** Zana Wangari Kiragu, Peter C. Rockers, Richard Neci Cizungu, Stephanie Ettinger de Cuba, Peresian Letayian, Judith Asin, Veronika J. Wirtz

**Affiliations:** 1 Department of Global Health, Boston University School of Public Health, Boston, Massachusetts, United States of America; 2 Ecumenical Pharmaceutical Network, Nairobi, Kenya; 3 Department of Health Law, Policy and Management, Boston University School of Public Health, Boston, Massachusetts, United States of America; 4 Consultant, Nairobi, Kenya; New Zealand Institute of Public Health, UNITED KINGDOM OF GREAT BRITAIN AND NORTHERN IRELAND

## Abstract

In many low- and middle-income countries (LMICs), faith-based organizations (FBOs) play a critical role in ensuring access to essential pharmaceutical services. Despite their longstanding presence in this role, there are few existing reviews describing the contributions of FBOs to pharmaceutical systems in LMICs. This study aims to provide a structured overview of the existing literature on FBO engagement in pharmaceutical systems across LMICs. This study utilizes the Pharmaceutical Systems Strengthening (PSS) Framework to categorize FBO contributions across key components of the pharmaceutical system. A scoping review, guided by the Arksey & O’Malley approach, was undertaken. Searches focused on English-language literature from 2000 onward across multiple databases. Two reviewers independently screened titles and abstracts, and relevant full texts were analyzed to extract the contributions of FBOs. We identified 524 papers, with 30 meeting inclusion criteria after eligibility assessment. These studies spanned 21 LMICs, 19 of which were in Africa, and documented FBO contributions across five religions: Christianity (N = 30), Islam (N = 4), Hinduism (N = 1), Sikhism (N = 1), and Buddhism (N = 1). While FBO engagement is noted in all pharmaceutical system components, the most documented FBO contributions were in: financing (donations, pooled procurement and drug revolving funds), regulatory systems (quality and safety surveillance), human resources (capacity building) and pharmaceutical products and services (operating outlets that supply pharmaceutical products, promoting increased uptake and adherence). Findings also highlighted that FBOs collaborate within and across sectors. However, there remains a paucity of data on evaluating FBO contributions. This scoping review highlights the diverse roles of FBOs in pharmaceutical systems across all components of the PSS framework to address critical system gaps. The overrepresentation of Christian FBOs in the literature underscores the need to further explore engagement across other religious traditions. A critical gap identified is the lack of rigorous measurement of FBO outcomes and impact.

## Introduction

The pharmaceutical system is a crucial part of a well-functioning health system. Several stakeholders, including faith-based organizations (FBOs), are important contributors to pharmaceutical systems, particularly in LMICs. FBOs are actors driven by religious ethics, with a long history of providing social services in alignment with their respective religious affiliations. One of the key driving factors of their involvement is the limited capacities of governments in LMICs to finance and deliver health services, which has translated to the inclusion of FBOs as donor-funding recipients [1].

FBOs comprise various bodies engaged in development activities, including non-governmental organizations (NGOs), civil society organizations, communities, and religious organizations [2]. Research into the role of faith/religious organizations in global health and economic development first emerged in the early 2000s in the midst of the Human Immunodeficiency Virus (HIV) epidemic, with a global lack of efficient healthcare institutions providing needed services. The bulk of the evidence base on the role of faith/religious organizations in the field of development and social service provision, including health, is found in gray literature [3]. While grey literature may not undergo peer review, it can be reliable, provided attention is paid to the quality of research, including the methodology and review status of reports. Furthermore, the field is growing, with an increasing number of publications, as seen in the Faith and Health series in The Lancet and in publications from faith-focused journals such as the Christian Journal of Global Health [4,5].

The growing literature provides evidence that FBOs play a role in the pharmaceutical systems of LMICs. For example, they supported responses to HIV/AIDS, COVID-19, and Ebola through medicine supply and advocacy for treatment adherence and vaccination. However, this does not accurately represent their comprehensive pharmaceutical functions. While some previous assessments have examined the role of FBOs in the pharmaceutical system, they have limitations [6–8]. First, none of these assessments used a pharmaceutical systems strengthening framework a priori to design the study [6–8]. Furthermore, these prior assessments were limited in geographic scope, primarily focused on Christian FBOs in Africa. In addition, two of the studies focused solely on the pharmaceutical supply chain, while the third focused exclusively on pharmaceutical service delivery, with none engaging in a holistic assessment of all pharmaceutical functions [6–9]. Therefore, this scoping review seeks to build on these prior assessments by providing a comprehensive overview of the literature on FBO contributions in the pharmaceutical systems of low-and middle-income countries.

Unlike other assessments, we apply a pharmaceutical systems strengthening (PSS) framework to guide our study [10]. This framework was designed to measure pharmaceutical system strengthening efforts over time and across countries. It was selected for this work due to the comprehensive and methodologically rigorous approach to its development. It provides sufficient depth to pharmaceutical functions while also considering both pharmaceutical supply chain and service delivery. In addition, beyond conceptualizing the scope of the pharmaceutical system, the framework measures the presence of appropriate structures and processes to support contributions to the pharmaceutical system, allowing us to move beyond describing the role to highlighting areas where FBOs might align with or diverge from best practices [10]. Since FBOs operate at all levels of the pharmaceutical system and constitute a substantive market share of health system, selection of a framework designed for national pharmaceutical systems is well-aligned [5,9,11]. Finally, the PSS is the most recent comprehensive framework and the only one focused explicitly on pharmaceutical system strengthening, aligning with our goal of understanding how FBOs contribute to pharmaceutical system performance [12].

We also include all religious groups in our assessment, unlike previous studies. Thus, our approach facilitates the generation of a comprehensive understanding of FBOs’ role and contributions to pharmaceutical systems. In doing so, this work addresses the research question: What pharmaceutical functions do FBOs play, and how do they contribute to the performance of a pharmaceutical system?.

## Methods

### Study approach

According to the Canadian Institutes of Health Research, scoping reviews are defined as “*exploratory projects that systematically map the literature available on a topic, identifying the key concepts, theories, sources of evidence, and gaps in the research”* [13]. Scoping reviews differ from systematic reviews in that authors usually do not assess the quality of included studies. They are typically undertaken in two scenarios: either the potentially relevant literature is likely to be vast and diverse in method, theoretical approach, or discipline, or insufficient literature exists for a full systematic review [14]. The topic at hand faces both issues to an extent. First, given their religious affiliations and history, examinations of FBOs, by nature, exist in religious studies, development studies, and public health. Second, the paucity of data quantifying FBO contributions to health services is well documented [11,15]. Thus, a scoping review of both peer-reviewed and gray literature is the best approach to provide an overview of the literature.

The Arksey and O’Malley Approach was selected to guide the scoping review, considering the refinements to this approach proposed by Levac et al. [13,14]. This approach highlights six key stages: 1) Identifying the research question, 2) Identifying relevant studies, 3) Study selection, 4) Charting the data, 5) Collating, summarizing, and reporting results, and an optional stage 6) Consultation with stakeholders [13,14]. The PRISMA 22-item checklist for scoping reviews was used to organize the write-up into introduction, methods, results, and discussion sections, as indicated in S1 Checklist [16]. Each of the stages of the Arksey and O’Malley approach and how they have been applied in this research are described in S1 Table. The scoping review protocol was retrospectively registered with the Open Science Framework (OSF). The registration is currently embargoed, and a view-only link can be accessed here: (https://osf.io/sdjvg/?view_only=acb7dd564d9e49fa933aa974198436a0).

### Search strategy

Various databases, including PubMed, Web of Science, Google Scholar, and Academic OneFile, were used for our search. Search terms to capture the key concepts “pharmaceutical systems,” “faith-based organizations,” and “low-and-middle-income countries” were identified and refined with librarian support. The search terms were refined to facilitate the identification of records from low, lower-middle, and upper-middle-income countries as defined by the World Bank in 2022 [17], with a publication date in the year 2000 and beyond, that discussed pharmaceutical functions, pharmaceutical services, or pharmaceutical care by FBOs, and published in English. Faith-based organizations were defined broadly to include NGOs, civil society organizations, communities, and religious organizations engaged in development activities, while pharmaceutical functions, services, and care were conceptualized using the PSS framework [2,10]. The selected databases were searched between July 8^th^ and 26^th^ 2024, with targeted searches continuing intermittently until December 2024 (Table 1). See S2 Table for more details on the search terms applied in each database.

**Table 1:** Search results from selected databases.

Database	Number of articles
PubMed	147
Google Scholar	75
Web of Science	55
Gale Academic OneFile	235
Targeted searches	12

### Study selection

After retrieving the records, they were uploaded to Rayyan software to organize the screening process [18]. Three reviewers were involved in the screening process. One reviewer manually resolved duplicates identified using Rayyan software. Two reviewers assessed the title and abstract of each record separately for relevance. Reviewers met at the beginning of the review process to discuss inclusion criteria and resolve any concerns about selecting studies [13]. The issues covered during the introductory meetings included the research question, describing the approach to the scoping review (Arksey and O’Malley framework), summarizing the conceptual framework guiding the review (PSS framework) and highlighting the inclusion criteria. These discussions ensured consistency between reviewers. The key inclusion criteria applied were:

- Records focused on low- and middle-income countries.
- Records published in the year 2000 and beyond.
- Records focused on FBOs addressing pharmaceutical services, care, outcomes, or functions as defined by the PSS framework.

All of the above criteria had to be met for a study to be included. Bi-weekly meetings were scheduled to discuss the screening progress, review independent decisions, and discuss disagreements. The comments function in Rayyan was also used to describe rationale of decisions, in the event meetings were impossible. To resolve disagreements, each reviewer presented their rationale for a given decision they made, and then a discussion followed. Following discussions and a review of comments, the review criteria were adjusted and clarified as needed, with consultation of a third party for guidance and to resolve disagreements where necessary. Additional studies for inclusion were identified through targeted searches of key authors, journal searches of interest such as the Christian Journal for Global Health, review of reference lists, and targeted searches of relevant organizations.

### Data extraction

**Fig 1:**
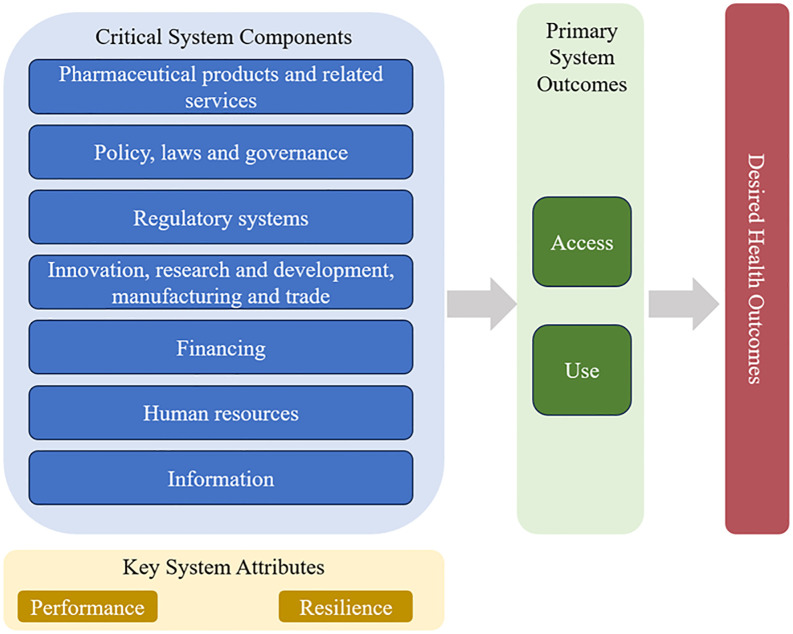
Pharmaceutical systems strengthening measurement framework. Brown SM, Wirtz V, Hafner T, Aboagye-Nyame F, Guzman J, Nfor E. *PSS Insight v 2.0: A Framework and Indicators for Measuring Pharmaceutical Systems Strengthening*. USAID Medicines, Technologies, and Pharmaceutical Services (MTaPS) Program; 2021.

Once included, one reviewer reviewed the full text to confirm alignment with the research question. If deemed relevant, they began the data extraction process. Charting of data included highlighting the pharmaceutical functions, attributes, and outcomes as defined by the PSS framework, which was used to provide a priori themes for this analysis (Fig 1) [10]. After reviewing the first 7 included records, an additional column (theme) focused on measurement was added to the data extraction table, to capture the extent to which various components were monitored or evaluated [10,14].

The collation and summarizing stage included numerical summary analysis and qualitative thematic analysis, reporting of results, and discussion of the implications of findings [14]. The numerical summary analysis described the number of studies included, types of study designs and/or publication formats, publication year, types of faith-based organizations studied, and countries of study [13]. Qualitative thematic analysis synthesized a priori themes from the PSS framework while discussing additional emergent themes.

#### Ethical declaration.

Ethical approval was not required for this scoping review because all the papers synthesized were already published and publicly available.

## Results

### Selection of sources of evidence

**Fig 2:**
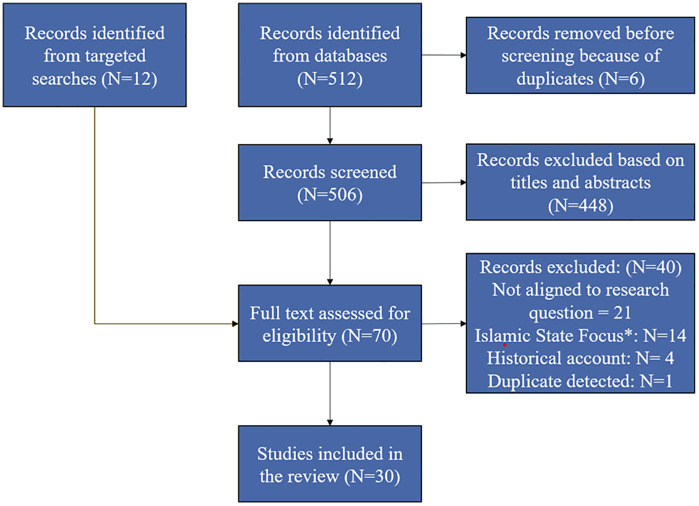
Selection flow-chart. Note: Papers focused on Islamic states were excluded because they fell beyond the study’s definition of FBO as detailed in the introduction.

The selection flow chart is laid out in Fig 2, resulting in a total of 30 papers being included in the review. During screening, there were 22 true disagreements, whereby one reviewer opted to include and another to exclude, or vice versa.

### Numerical summary analysis

A total of 30 articles were included in the analysis (S3 Table). These papers had 5 different study designs/publication formats: cross-sectional study (N = 15), case study (N = 7), commentary (N = 4), systematic review (N = 2) and a thematic literature review (N = 1). One paper had two study designs (a thematic literature review and a cross-sectional study). The papers focused on 21 LMICs, with the most frequently studied countries being Cameroon (N = 10), Kenya (N = 10), Uganda (N = 8), Nigeria (N = 8), Ghana (N = 7) and DRC (N = 7). Except India and Haiti, all countries were in Africa. The religious group of focus was primarily Christianity, whereby all 30 papers considered Christian FBOs. Four papers also addressed Islamic FBOs and only one addressed Sikh, Hindu and Buddhist faith actors. The funding sources of the included papers varied including public sponsors (N = 7), faith-based sponsors (N = 4), multi-sectoral sponsors (N = 4), non-governmental sponsors (N = 2) and industry sponsors (N = 2). In addition, 4 papers were not sponsored while 7 did not report on their funding sources (S3 Table).

### Thematic analysis

A matrix was developed to map the role of FBOs in the pharmaceutical system as defined by the PSS framework (Table 2), and quotes from the papers were included in S1 Text. Analysis revealed that each of the 7 pharmaceutical system components in the PSS framework were engaged in by FBOs, with the most frequently documented components being financing (N = 13) focusing on how funding flows through FBOs into the pharmaceutical system and ultimately reaches the patient and regulatory systems (N = 11) addressing quality & safety surveillance. The frequency of the additional components addressed was as follows: human resources (N = 10), pharmaceutical products and related services (N = 9), information (N = 7), policy, laws and governance (N = 4), and innovation, research and development (R&D), manufacturing and trade (N = 1) (Table 2).

**Table 2:** Pharmaceutical system components.

Reference	Pharmaceutical System Components						
	Pharmaceutical Products & Related Services	Policy, laws & governance	Regulatory Systems	Innovation, R&D, Manufacturing & Trade	Financing	Human Resources	Information
Soni et al. 2023							
Syed et al. 2023							
Azmat et al. 2019	Procurement, Distribution						
Petersen et al. 2017			Quality & safety surveillance		Costing & pricing (subsidized price)	Development	
Khuluza et al. 2017			Quality & safety surveillance				
Gnegel et al. 2022			Quality & safety surveillance				
Domfeh et al. 2021	Procurement		Quality & safety surveillance		Resource coordination, allocation, distribution & payment (pooled procurement)		
Domfeh et al. 2021	Pooled procurement				Resource coordination, allocation, distribution & payment (pooled procurement)		Data collection, processing and dissemination
Joshi 2017							
Ntani et al. 2022						Development, Management	
Kilonzi et al. 2024					Resource coordination, allocation, distribution & payment (funder)	Development	Data collection, processing & dissemination (digital systems)
Velavan, 2012						Development	
Barden-O’Fallon, 2017							
Ayissi et al. 2012							
Ogembo et al. 2014						Development, Management	
Masselle AY et al., 2001	Selection						
EPN, 2018			Quality & safety surveillance				
EPN, 2020					Resource coordination, allocation, distribution & payment (drug revolving fund)	Development	Dissemination
Metzger et al. 2017		Coordination & leadership			Resource coordination, allocation, distribution & payment (donations, out of pocket payments)		Data collection and processing
Jalloh et al. 2024			Quality & safety surveillance (testing)		Resource coordination, allocation, distribution & payment (donations)	Development	
Gabel et al. 2024			Quality & safety surveillance (testing)				Data collection, processing and dissemination
Gnegel et al. 2020			Quality & Safety Surveillance (testing)				
Joshi et al., 2018		Coordination & leadership			Resource coordination, allocation, distribution & payment (donations, diversifying funding sources)		
CCIH, 2015	Procurement		Quality & safety surveillance (good storage practices, pharmaco-vigilance)		Resource coordination, allocation, distribution & payment (patient fees)		
Institute for Reproductive Health, 2011		Policies (Advocacy)					
WHO, EPN, 2006	Selection, Procurement, Distribution	Coordination & leadership	Quality & safety surveillance (pharmaco-vigilance, testing)	Manufacturing	Resource coordination, allocation, distribution & payment (donations, drug revolving funds)	Development	Use of information for decision making (drug information services, computerized inventory control systems)
Ruark et al., 2021							
Mwenda, 2007	Selection		Quality & safety surveillance (testing)		Resource coordination, allocation, distribution & payment (Drug revolving funds, Donations)	Development	Data collection, processing & dissemination (Computerized systems)
Jaguga, 2018	Procurement				Resource coordination, allocation, distribution & payment (Pooled procurement)		
Budge-Reid et al., 2012	Procurement, Distribution				Costing & pricing (Free or subsidized medicines)	Development, Policy & strategy	

### Pharmaceutical system components

As defined in the PSS framework, the pharmaceutical products and related services component include the following elements: selection, procurement, distribution and use. The policy, laws and governance component includes the following elements: policies, laws and regulation, coordination and leadership, ethics, transparency and accountability. The regulatory systems component includes the following elements: product assessment and registration, licensing of establishments and personnel, inspection and enforcement, quality and safety surveillance, regulation and oversight of clinical trials and control of pharmaceutical marketing practices. The innovation, R&D, manufacturing and trade component includes the following elements: innovation, research and development, manufacturing capacity and intellectual property and trade. The financing component includes the following elements: resource coordination, allocation, distribution and payment, financial risk protection, revenue and expenditure tracking and management and costing and pricing. The human resources component includes the following elements: policy and strategy, management and development. The information component includes the following elements: information policy and data standardization, data collection, processing and dissemination and use of information for decision making [10].

#### Component 1: Financing.

The PSS framework defines financing broadly as the management of resources to ensure sufficient and sustained funding for the functioning of the pharmaceutical system, including the purchase of pharmaceutical products and related services [10]. The literature discussed financing in two ways: how funding flows through FBOs into the pharmaceutical systems of LMICs and how FBOs contribute funding to the pharmaceutical system.

Findings indicated that FBOs have diverse funding sources which enabled them to provide pharmaceutical products and services. These included faith-based or secular donors, governments and financial returns from their operations (drug revolving funds) [6,7,19–22]. The primary funding sources of FBOs varied by the level at which they operate within the pharmaceutical system and the degree of their establishment.

At the national pharmaceutical supply level, drug supply organizations (DSOs) across multiple countries typically received an initial donation in the form of funds or pharmaceutical products from a donor during start up, which they then supplied at a small profit margin to facilitate financial returns [6,7,21]. As the DSOs became more established, they were able to rely more on returns from pharmaceutical product sales to purchase more products, while simultaneously applying for and obtaining more faith-based and secular donor funds to supplement their functioning [7,20]. Therefore, at the national level, once FBOs became established, they were more likely to generate their own financing and reinvest it directly into the pharmaceutical system, particularly through the purchase and supply of pharmaceutical products [7].

The primary mechanism through which national-level FBOs that are established and able to sustain themselves through medicine sales is called a drug revolving fund (DRF). This is a system whereby an initial amount of capital is invested (typically using donor funds) to purchase pharmaceutical products, and the products are sold at a cost that provides returns that can be reinvested back into purchasing more products [6,21,23].

At the service delivery level, FBOs were mainly described as conduits through which funding flowed into the pharmaceutical system from external sources. Funding sources varied across countries and by pharmaceutical product [7,8,20,23–25]. One study postulated that patient fees were the primary funding source of African faith-based facilities, with additional sources including external donors and government subsidies [24]. For instance, with some pharmaceutical products, such as contraceptives, faith-based facilities relied on the government for the supply since they are provided as part of national programs [24].

Given the reliance on funding from external sources, FBOs have strived to establish resource management techniques that can save costs and ensure the sustainability of their pharmaceutical system functioning. Three key resource management approaches that are evident in the literature are: DRFs(6,21,23) (previously described), pooled procurement programs [22,26,27], and diversification of funding sources [28].

The FBOs’ ability to engage in one or more of these approaches depended on relationships with other stakeholders in the pharmaceutical system (such as donors), the country context, and the type of pharmaceutical product in question. For instance, as previously described, DRFs are evident and functioning in the Kenyan drug supply organizations operating at the national level [22]. Furthermore, at the regional level, the Ecumenical Pharmaceutical Network (EPN) has had success in diversifying its funding sources to facilitate sustainability [28].

The funding source is crucial because it influences how FBOs price their pharmaceutical products and services. This was observed particularly with donations, where restrictions were placed on how FBOs priced the products received from donors [6,25]. For instance, faith-based facilities were not permitted to charge for contraceptives received for free from the Ministry of Health, but some were permitted to charge for procedures related to the contraceptive such as insertion of intrauterine devices (IUDs) [24]. In a similar vein, 38% of DSOs (5 out of 16) assessed in one study reported that drug donations negatively affected their operations [6]. The main reason cited for this was suboptimal coordination between the donors and DSOs, translating to inappropriate donations, such as short-expiry medicines, medicines that did not align with contextual needs, insufficient or irregular quantities of medicines and damaged or compromised packaging [6]. This interfered with existing supply chain, storage and distribution processes, and had cost implications [6]. Unfortunately, none of the DSOs experiencing such challenges reported them to the World Health Organization (WHO) or their donor, due to concerns about compromising long-standing relationships and excluding themselves from future financial support [6].

Another approach that FBOs have taken, as described in the literature, is the pooled procurement program, which involves joint purchasing to mitigate exorbitant pricing [22,26,27]. Specifically, FBOs pool their capacity to facilitate the sourcing of larger quantities of pharmaceutical products at lower costs [26]. Pooled procurement at the national level was noted to be successful for FBOs, with substantive cost reductions observed [26].

However, the benefits of pooled procurement on the regional level have been less apparent, with challenges surrounding the harmonization of regulatory requirements across countries [22]. For instance, an assessment of the East Africa Pooled Procurement Mechanism that engaged faith-based DSOs from Kenya, Uganda, Tanzania and Rwanda reported major, moderate and minor challenges, including conflicting regulations across the different countries and limited financial resources to procure pharmaceutical products [22].

Alongside these mechanisms to manage resources sustainably, the ethos of serving the marginalized that underlies FBO functioning remained apparent. Specifically, FBOs were described as providing products at lower costs or free of charge, in alignment with patients’ ability to pay [7,8]. Notably, one study reported that 9 out of 10 faith-based facilities provided free or subsidized medications to the poorest patients [8].

#### Component 2: Regulatory systems.

This component’s key focus is on ensuring the safety, efficacy and quality of pharmaceutical products and related services [10]. Most of the papers describing the role of FBOs under this component touched on quality and safety surveillance, specifically using the “Minilab”. This is a technique developed by the Global Pharma Health Fund (GPHF) and implemented by the German faith-based NGO German Institute for Medical Mission (DIFAEM) and the Ecumenical Pharmaceutical Network (EPN) in 2010 to support Drug Supply Organizations in LMICs with quality assurance [29,30].

The GPHF Minilab uses thin layer chromatography to detect products with less than 80% of the active pharmaceutical ingredients. It is equipped to test for the presence of approximately 100 pharmaceutical ingredients and to test for the disintegration of tablets. It is a cheap, simple, easily portable approach that precludes the need for a fully equipped laboratory, which are often not readily available in LMICs, more so in rural areas [19,30–33]. Implementing the Minilab project within faith-based drug supply organizations has worked well, because it leverages the different levels of capacity of various DSOs within the Minilab network. For instance, if a sample fails testing within a Minilab partner after two tests, it is sent for pharmacopeial testing at the World Health Organization prequalified laboratory at the Mission for Essential and Drug Supplies (MEDS), one of the Minilab partners in Kenya [30,32,33].

The value of the faith-based Minilab Network on quality assurance is evident in three ways. First, it has facilitated detection of substandard products and their recall from the market [30,32]. Second, it has, in some cases, provided evidence refuting some of the exaggerated claims on the prevalence of substandard and falsified medicines, thus rebuilding the public’s trust in medicines provided in the public and faith-based sectors [31]. Third, it is worth noting that while the Minilab kits have been distributed by GPHF across multiple countries since 1998, the DIFAEM/EPN Minilab Network that has been actively disseminating their results for shared learning [19,30–33]. Of note, Dr. Richard Jaehnke, the “father” of the Minilab stated: *“I find with church groups, there is more of a rapport – […] and it´s more transparent. […] They track fake medicines down even more effectively than the police – because in their eyes, delivering counterfeit medicine with nothing inside is ´like cheating God’.”* [29].

Beyond quality testing, papers revealed that FBOs ensure good storage of pharmaceutical products, safeguarding the quality and safety of the products. [6,25]

#### Component 3: Human resources.

The human resources component includes all activities involved in ensuring adequate numbers of appropriately trained human resources to manage and supply quality pharmaceutical products and services [10]. Key issues addressed in this component included FBOs contribution to human resource development, management, policy and strategy [6–8,19,21,23,34–36]. The faith-based sector plays a critical role in the capacity building of pharmaceutical staff. This includes providing resources for training of pharmaceutical staff online and in person, as well as training on specific projects or techniques, such as the Minilab Network. Minilab has allowed pharmacists and pharmacy technicians to build capacity in quality assurance techniques, thus expanding the capacity of the pharmaceutical workforce on quality surveillance.

In Cameroon, the Cameroon Baptist Convention Health Services (CBCHS) responded to the lack of pharmacists by providing a three-year formal training for pharmacy technicians to provide patient-oriented services [36]. They started the training program in 2003, and their 2019 annual report cited that more than 800,000 patients had received care from the CBCHS pharmacy department [36]. An assessment of this program revealed that 4 out of 5 healthcare providers were generally satisfied with the patient-oriented services provided by pharmacy [36]. However, there were some concerns about their ability to engage in bed-side discussions and assist doctors in drug selection, with only 1 out of 5 providers being satisfied with their role in this [36].

These innovative approaches to addressing human resource needs are also evident in other contexts, whereby the Christian Social Services Commission (CSSC) in Tanzania implemented a project to expand clinical pharmacy services in faith-based and public hospitals in Tanzania [20]. The project, which was rolled out in collaboration with Muhimbili University of Health and Allied Sciences (MUHAS) and the German non-profit action medeor, trained 104 pharmacists working in hospitals at different levels of the health system [20]. A checklist system was used to assess the implementation of clinical pharmacy services in the facility and included various indicators such as presence of a ward round schedule for pharmacists and presence of a focal person for clinical pharmacy services [20].

In Kenya, the Mission for Essential Drugs and Supplies (MEDS) has also implemented several training programs focused on governance, strategic management and health commodity management, having trained over 40,000 professionals including health care workers, health systems managers, administrators and auxiliary staff in faith-based, public and private sectors [23].

On a regional level, The Ecumenical Pharmaceutical Network has also implemented efforts to expand the pharmaceutical workforce. Through their Ecumenical Scholarship Program (ESP), scholarships have been awarded to 21 pharmacy staff (14 diplomas, 7 degrees) from 21 faith-based facilities across 8 countries as of 2020 [23]. Insights from our consultation with stakeholders revealed that an additional 21 scholarships are being provided for 2023–2026. Furthermore, they have an online Essentials of Pharmaceutical Practices (EPP) training course tailored to increase the capacity of hospital-based pharmaceutical staff. Noting that improving pharmaceutical practice is not sufficient to influence system outcomes, EPN has also developed several online courses in partnership with MEDS to address topics including leadership and governance and financial management [23].

While efforts to build capacity at the national and regional level are evident, gaps persist at the service delivery level. For instance, an assessment of 363 church facilities in five African countries revealed that only half of them had qualified pharmaceutical personnel [8]. This same assessment noted that while capacity building activities were undertaken, there was a lack of monitoring and evaluation to determine the outcome of the training, and reasons for its effectiveness or lack thereof [8].

#### Component 4: Pharmaceutical products and related services.

This component lies at the core of the pharmaceutical system, and includes the processes of selection, procurement and distribution of pharmaceutical products, as well as systems to ensure appropriate use by providers (prescribing) and end users [10]. Findings revealed that FBOs operate pharmaceutical outlets which are engaged in the selection, procurement, distribution and use of pharmaceutical products [6,8,21,22,25–27,37,38]. These outlets exist at different levels of the pharmaceutical system.

One study reported that of the 363 faith-based facilities they assessed, 55% had written standard operating procedures (SOPs) for procurement and 68% of these implemented them [8]. Another study reported that the faith-based DSO in Kenya – MEDS – reported an increase in its formulary from 50 products in 1986–1500 products in 2006, indicating its increasing role in selecting pharmaceutical products for distribution [21]. To facilitate procurement, FBOs quantify medicines using multiple approaches, including sales data from prior periods, customer requests, and morbidity patterns [6].

In addition, FBOs typically use the national essential drug list or WHO Essential Medicines List to select products for their formularies [6]. For instance, an assessment of church-owned facilities in Tanzania revealed that 87% of pharmaceutical products were prescribed according to the country’s essential medicines list [38]. The selection from these tools is made by drug committees, procurement teams or individual decisions pharmacists/doctors working at the FBO [6]. Drug therapeutics committees were evident at the service delivery level with one study reporting 57% of assessed facilities had one [8]. Faith-based DSOs have also collaborated in pooled procurement efforts [22,26,27]. However, the joint bulk purchasing efforts at regional levels did not yield the expected benefits of increased availability and affordability [22].

Regarding distribution, faith-based DSOs typically offer delivery services to their clients, with the flexibility of allowing their customers to make their own arrangements in a preferred manner [6]. One study focused on distribution in the humanitarian space revealed that faith-based humanitarian organizations had slightly different priorities than secular humanitarian organizations. Specifically, FBOs perceived the culture of beneficiaries, that is, traditions, language, food, clothing, and norms, to be a more important factor than the religion of beneficiaries in the successful implementation of humanitarian supply, whereas the opposite was true for secular organizations. This is because the secular FBOs are typically faced with more challenges in their humanitarian supply chains when they are dealing with religious beneficiaries, due to mistrust since beneficiaries preferred to engage within their faith groups, suggesting that the religious identity of FBOs offered benefits in terms of customer preference [37].

#### Component 5: Information.

This component speaks to the generation and dissemination of timely, reliable information to support decision making and pharmaceutical system functioning [10]. The primary role that FBOs take in pharmaceutical information systems is through data collection, processing and dissemination [6,20,21,23,26,33]. For instance, FBOs contribute to the dissemination of pharmaceutical information online through print newsletters and online training content to strengthen capacity of pharmaceutical staff [6,23,29].

Digitized systems to collect pharmaceutical system data are present, though not yet widely implemented across all levels of the pharmaceutical system and deemed to be an area for improvement [6]. Among FBOs functioning at the national level such as DSOs, digitized information systems are more pervasive [6,21].

At the service delivery level, digitization of information systems is still expanding, though this is the case in both the faith-based and public sectors, as detailed by one study where both public and faith-based facilities were engaged in a clinical pharmacy intervention that included access to digital information systems [20]. In fact, one faith-based DSO in Ghana reported the challenges of no streamlined information system with its clients (faith-based facilities), which prevented real-time access to requisite data to inform their supply chain [26].

This gap in internal systems to facilitate data collection for supply activities, including forecasting demand, was included as a recommendation for faith-based organizations engaged in the reproductive health supply chain, to strengthen their engagement with the public sector reproductive health supply chains [24].

#### Component 6: Policy, laws and governance.

This component represents the hub of coordination for the pharmaceutical system, providing frameworks, systems and structures to coordinate pharmaceutical system activities and achieve system goals [10]. It is evident from the literature that FBOs contribute to coordination and governance of pharmaceutical services, primarily due to their structural set up [24,28]. FBOs exist within membership networks that facilitate institutionalization of various policies and coordination of pharmaceutical services from supply chains at the national level to service delivery at the health facility level [6,24,28]. Christian faith-based facilities typically belong to a Christian Health Associations (CHAs) and are typically the client base of faith-based DSOs [6,7,34]. CHAs and DSOs are typically members of a regional faith-based network, such as the Ecumenical Pharmaceutical Network. These relationships facilitate coordination and joint advocacy efforts with various stakeholders in the pharmaceutical system, including patients, to address challenges related to access to pharmaceutical products [28].

The power of their coordinated efforts is evident in Cameroon, where seven FBOs came together to address the challenge of stockouts of family planning products [24]. Together, they established objectives and registered their alliance with the government, to facilitate joint efforts towards reducing stockouts at their facilities [24].

Furthermore, some FBOs are engaged in advocacy to shift policies around family planning, both internal to their organization and externally at the national and international level [39].

#### Component 7: Innovation, research and development and manufacturing.

FBOs’ contribution to this component is only described in one paper, where they cite engagement by six out of 16 assessed DSOs in manufacture of products [6]. Of these six DSOs, three of them shared the different types of pharmaceutical products they produced, including syrups (quinine), topical lotions and ointments (calamine), pessaries and suppositories (analgesics and anti-hemorrhoid), various anti-infective tablets, eyedrops and infusions [6].

### Pharmaceutical system attributes: performance and resilience

Pharmaceutical system attributes include the performance (efficiency, quality, safety and responsiveness) and resilience (aware, diverse, self-regulating, integrated and adaptive) of the pharmaceutical system [10]. The performance and resilience of FBOs in the pharmaceutical system was not explicitly addressed at length in the literature, except for two papers, one which qualitatively described the efficiency of an FBO venture (pooled procurement program) [26,40] and another which mentioned the presence of disaster and preparedness plans at FBOs, indicating awareness and preparation for potential health threats (Table 3) [8].

**Table 3:** Pharmaceutical system attributes and outcomes.

Reference	Pharmaceutical System Attributes		Primary System Outcomes	
	Performance	Resilience	Access	Use
Soni et al., 2023			Acceptability	Consumption
Syed et al., 2023			Acceptability	Dispensing, Consumption
Azmat et al., 2019				
Petersen et al., 2017				
Khuluza et al., 2017				
Gnegel et al., 2022				
Domfeh et al., 2021			Availability	
Domfeh et al., 2021	Efficiency			
Joshi, 2017			Acceptability	Consumption
Ntani et al., 2022				
Kilonzi et al., 2024				
Velevan, 2012				
Barden-O’Fallon, 2017			Availability, Acceptability	Prescribing, Dispensing
Ayissi et al., 2012			Acceptability, Affordability	Dispensing, Consumption
Ogembo et al., 2014			Affordability	Dispensing, Consumption
Maselle AY et al., 2001			Acceptability	Prescribing
EPN, 2018				
EPN, 2020			Availability	
Metzger et al., 2017			Availability, Affordability	
Jalloh et al., 2024			Availability	
Gabel et al., 2024				
Gnegel et al., 2020				
Joshi et al., 2018				
CCIH, 2015				
Institute for Reproductive Health, 2011			Acceptability	
WHO, EPN, 2006			Availability	
Ruark et al., 2021				Consumption
Mwenda, 2007				
Jaguga, 2018				
Budge-Reid et al., 2012		Aware (Disaster preparedness plans)		Prescribing, Dispensing

As defined in the PSS framework. Performance includes efficiency, quality, safety and responsiveness. Resilience includes being aware, diverse, self-regulating, integrated and adaptive. Access includes availability, affordability, acceptability and accessibility. Use includes prescribing, dispensing and consumption [10].

### Pharmaceutical system outcomes: Access and use

Pharmaceutical system outcomes include access (availability, affordability, acceptability, and accessibility) and use (prescribing, dispensing, and consumption) of pharmaceutical products [10]. The contribution of FBOs towards pharmaceutical outcomes was described in 6 papers, but only one presented a robust measurement of these outcomes (Table 3). The papers’ study designs/publication formats were case studies [41], commentaries [39,42] and cross-sectional studies [38,40] that addressed the scale and reach of FBOs, as measured by the outputs of their activities. However, there was one systematic review that included a cluster randomized control trial evaluating an FBO’s implementation of a chronic disease self-management tool that addressed medication adherence [43].

The most prominent contribution that FBOs are described to make towards pharmaceutical system outcomes is that of acceptability (a component of access) and use. For instance, in one case study seeking to address the challenge of vaccine hesitancy through engaging FBOs and leaders in a communication strategy, FBO leaders were able to increase confidence in vaccines, and ultimately increase uptake [41]. Furthermore, as described in another commentary, FBOs’ role in addressing hesitancy is also evident in the context of family planning products [42].

The critical role of FBOs in increasing uptake and adherence to pharmaceutical products is attributed to their ability to interact with the community and ensure cultural appropriateness in any pharmaceutical programs or interventions. Therefore, FBOs’ were perceived to be critical for sustained engagement of communities with requisite pharmaceutical services. Again, it is important to note that these are descriptive studies that measured the outputs of FBOs’ programmatic efforts to increase uptake and adherence to pharmaceutical products. Some terms used to describe FBO contributions to pharmaceutical system outcomes include “ownership of vaccine programs”, “sustained participation of FBOs” and “sustained engagement” [41,43].

As an example, FBOs are well-positioned to influence reproductive health behavior and choices amongst their communities and can do so through sensitization campaigns [40,44]. One such sensitization program on the Human Papilloma Virus (HPV) vaccination was successful in achieving high levels of interest in getting vaccinated (76.3%), high levels of recommending the vaccine to friends/relatives (73.9%), and ultimately increased uptake of the HPV vaccine (34%) [40].

It is worth noting that while FBOs were willing to increase the acceptability and use of pharmaceutical products, some differences existed related to the audience to whom products and services should be directed. For instance, FBOs generally do not agree with providing family planning services to young, unmarried people [39]. These differences can sometimes pose challenges for secular actors as they collaborate with FBOs to address the uptake of family planning products [39].

FBOs also contribute to the availability of pharmaceutical products, with strategies such as pooled procurement programs and DRFs being implemented to facilitate access to sufficient quantities of pharmaceutical products [23,27]. Assessment of the availability of products at faith-based facilities revealed that products were generally available, with one paper citing a 90% chance of church facilities having chloroquine and 70% chance of having penicillin in stock. (ref Jalloh, 2024). Another study noted that twelve of the 16 reproductive health products were used by at least 80% of the FBO facilities in their assessment [24]. However, availability of certain product types (contraceptives) at the service delivery level (faith-based facilities) were reported to be lower in the faith-based sector when compared to other sectors [45].

At the national level, while DSO customers were generally appreciative of the quality and price of pharmaceutical products they received, they noted that availability at DSOs was suboptimal, with poor order fill-rates. Therefore, customers recommended that DSOs increase the range and quantities of products stocked to improve availability [6].

While no papers formally measured affordability, it was worth noting that the costing of pharmaceutical products was discussed, and there was an indication that the cost depended on the source of the product. Generally, FBOs charge a fee for pharmaceutical products to cover costs associated with customs clearance for donated products and facilitate the procurement of additional pharmaceutical products (drug revolving funds) [23,34]. In some cases, when they obtain pharmaceutical products free of charge from the government, they are required to dispense the products at no cost as well, but are allowed to charge for any related services, such as implant insertion [24].

### Additional pharmaceutical system themes

Beyond the PSS framework, two additional themes emerged from the synthesis of the papers: measurement and collaboration. First, it is evident from many papers that FBOs have well-established systems to measure the outputs of their efforts, such as the number of people trained during capacity-building efforts [23] and the number of samples failing quality assurance testing [19,30,33]. However, there appears to be less rigorous measurement of the effectiveness of their functions. This might relate to the ethos driving FBO work, which may lead to emphasis on less tangible aspects such as spiritual well-being and relationships [39].

This does not mean that no efforts are being made towards the measurement of outcomes. For instance, there are some qualitative assessments to evaluate the satisfaction of healthcare providers with FBOs’ pharmaceutical training efforts [36] and efficiency of pooled procurement programs [26]. In addition, two papers estimated the availability of family planning products and services at faith-based facilities [24,45].

Collaboration within the faith-based sector and beyond the sector is also evident across most of the papers. Due to the structure of faith-based organizations within networks, they typically engage with a variety of partners within the faith-based sector. For instance, CHAs work closely with their affiliated health facilities, while DSOs across different countries work towards common goals, as was seen through the East African Pooled Procurement Program [22]. Furthermore, FBOs based in high income countries collaborate with those in LMICs through providing funding (e.g., DIFAEM and Bread for the World) [28]. FBOs also often engaged with government actors, either by receiving pharmaceutical products from government medical stores [24], through implementation of programs in both public and faith-based facilities [20] or through advocacy to ensure inclusion of FBOs in public sector initiatives [24].

## Discussion

This scoping review describes the various functions of FBOs within the pharmaceutical system, guided by a comprehensive pharmaceutical systems strengthening framework. Using numerical summary analysis and thematic analysis, the review captures both the extent and nature of FBO engagement in the pharmaceutical system, as evidenced by the number of papers mentioning contributions to specific pharmaceutical system components, attributes, or outcomes and the description of activities within each of these domains. The findings indicate that FBOs address key pharmaceutical system challenges, including a lack of qualified pharmaceutical personnel and the prevalence of substandard and falsified medicines in LMICs. Furthermore, they contribute to the financing and provision of pharmaceutical products and related services, a critical role facilitated by the trust the communities they serve have in them.

This review also provides insight into the extent of FBOs’ engagement in the pharmaceutical system across different religious groups. In addition, the review reveals that Cameroon and Kenya are the most studied LMICs, likely relating to the critical and expansive role FBOs play in these respective health systems [1,46]. It therefore contributes to the existing body of knowledge through incorporating a comprehensive lens that considers all components and levels of the pharmaceutical system and the diverse religious affiliations of FBOs [6,7].

As a trusted sector, FBOs are critical in channeling funds into the pharmaceutical system and supporting the public sector in ensuring the quality and safety of pharmaceutical products. FBOs’ contributions to quality and safety surveillance are important, given the challenge of low-quality medicines across LMICs—the World Health Organization (WHO) reports that approximately 1 in 10 pharmaceutical products available in LMICs are substandard or falsified [47]. The faith-based sector has emerged as a convenient channel for implementing quality testing technologies such as Minilab, which allows for surveillance in ruralg areas [19,30]. This is because of FBOs’ well-structured networks, with different types of organizations operating at multiple levels of the pharmaceutical system, which facilitate coordination across numerous African countries [48]. Furthermore, their commitment to quality and safety surveillance can also be tied to their religious ethics, where the manufacture and distribution of falsified medicines is perceived to be akin to “cheating God” [49].

Some national regulatory authorities already incorporate the low-cost technology Minilab into post-market surveillance, but these authorities still often face resource constraints [30,50,51]. Nonetheless, there is resistance to FBO engagement in quality surveillance, with some regulatory authorities citing concerns that FBOs are overstepping the government’s mandate [30]. To navigate this, FBOs need to continue building their advocacy capacity to ensure strategic, evidence-based framing of their value proposition, thus facilitating governments’ understanding of their role as a complement to national and regional quality and safety surveillance efforts.

The value of FBOs is also evident in their contributions to addressing pharmaceutical human resource gaps. Across the African continent, the lack of qualified pharmaceutical personnel is stark, with an average of only 0.4 per 10,000 population, compared to the global average of 4.8 per 10,000 [52]. FBOs have responded by incorporating capacity-building initiatives into their programming, thus reinforcing their broader role in strengthening the pharmaceutical system. Despite these efforts, persistent human resource gaps were noted in faith-based facilities [8], indicating that this is an area that warrants continued prioritization and attention by both faith actors and governments.

In addition, the operation of FBO outlets to provide pharmaceutical products and related services aligns with other studies which note that FBOs comprise a substantive market share of national health systems as estimated using health facility data [15]. Therefore, collaboration across public, academic and faith-based sectors, as modelled through the recently convened Georgetown-Lancet Commission on Faith, Trust and Health, is critical to ensure that the value proposition of the faith sector as it pertains to health systems is not only understood, but also aligned with broader national health priorities [53].

Despite their demonstrated value, FBOs have important sustainability challenges driven by shifting donor landscapes and the need to balance income generation with their mission to serve the poor. This study highlights that most FBOs in LMICs operate with mixed funding models, drawing from diverse sources shaped by country-specific contexts, organizational maturity, and the pharmaceutical products in question. This finding aligns with other studies, where FBOs are described to have a complex funding mechanism that has included a consistent substantial share of development assistance for health [54]. Specifically, it is reported that from 1990 to 2013, FBOs received 30% of the total development assistance for health in the non-governmental space [54].

FBOs’ reliance on external funding has meant that they must invest in advocacy with actors across sectors to ensure continued inclusion in funding from the government or other external donors, as they are essentially competing with public-sector facilities for resources [24,25]. FBO inclusion can be difficult to establish, with one qualitative study on the Cameroonian faith-based sector describing a complex relationship between the government and faith sectors, where faith-based actors reported feeling invisible and excluded by the government [55]. These complex relational dynamics could benefit from further exploration to facilitate improving trust and establishing sustainable faith-health partnerships [53].

Furthermore, reliance on external funding leaves FBOs vulnerable to foreign aid patterns, making it critical that FBOs explore strategic pathways to ensure their long-term sustainability within the pharmaceutical system [56]. Of note, foreign aid declined in 2024 for the first time in five years — a change subsequently exacerbated by the United States government’s foreign aid cuts in 2025 [57,58]. While FBOs have been adversely affected by these recent US funding cuts, faith-based stakeholders consulted during this scoping review also recognize this as an opportunity to speed up the process of identifying more sustainable funding pathways.

In contrast to the documented roles in financing, regulatory systems, human resources, pharmaceutical products, and related services, there is less evidence in the literature of FBO engagement in other components such as information, policy law and governance and innovation research and development. This evidence gap is likely shaped by the broader pharmaceutical ecosystem and aligns with other literature that reports FBOs spend more on field operations or more direct service delivery, rather than research and development [54]. Therefore, it would be worthwhile to gather quantitative and qualitative empirical data from multi-sectoral pharmaceutical system stakeholders, to understand how these broader dynamics, including perceptions and influence of other sectors, might shape how FBOs contribute to these components of the pharmaceutical system.

An evidence gap was also apparent in the limited assessment of FBOs’ effectiveness in their engagement in the pharmaceutical system relative to other sectors, with only three papers discussing such comparisons [31,37,45]. While the literature clearly describes what FBOs are doing in the pharmaceutical system, there is a paucity of evidence that measures the effectiveness of their contributions. Specifically, while outputs from their programming and activities are reported, outcomes and impact remain less apparent.

In alignment with this, the performance and resilience attributes are not comprehensively covered in the literature, and access and use as pharmaceutical outcomes are only described, not assessed. This aligns with the stated need for enhanced monitoring and evaluation among faith actors and more careful consideration of how to measure FBO efforts [3,55]. Measurement gaps might be attributed to the lack of proper assessment tools and agreed-upon benchmarks to measure best practices in pharmaceutical supply and management operations run by FBOs [6]. Strengthening measurement to ensure rigor is a critical area of improvement for the faith sector, in order to optimize their functioning and inform advocacy efforts that demonstrate their value proposition. To this end, faith actors might consider partnering with academic institutions to adapt PSS indicators for survey design to assess the current state of their pharmaceutical functions, or to apply more targeted outcome measurement tools such as the World Helath Organization/Health Action International availability and affordability tool [59].

Another notable gap in existing literature is the minimal coverage of other religious groups, with Christian FBOs being overrepresented. This might explain why prior assessments of FBOs in pharmaceutical systems have not considered religious groups beyond Christianity [6,7]. This over-representation in the literature may be in part reflective of historical donor dynamics, specifically with the United States President’s Emergency Plan for AIDS Relief (PEPFAR), which prioritized engagement with faith communities, particularly Christian FBOs, and likely contributed to their prominence in practice and documentation [60,61].

However, this review’s inclusion of other religious groups highlighted an interesting dynamic: in Islamic states, there is conflation between government and faith-based sectors. As a result, our search retrieved papers focused on Islamic States, which were ultimately excluded because they fell beyond our definition of FBOs [2]. Nonetheless, these findings highlight a potential area for future research on pharmaceutical systems engagement across different religious and governance contexts. This might include studies that examine specific religious groups independently, for instance focusing on Islamic groups to mitigate the risk of Christian groups dominating the narrative.

This scoping review has several limitations. First, being a scoping review, it did not assess the quality or rigor of the papers included. This means that while we can map out FBO contributions, we cannot evaluate the strength of evidence supporting each identified role or function. Second, some of the included papers are dated, particularly those discussing innovation, R&D, manufacturing, pharmaceutical products, and related services. Given the evolving nature of pharmaceutical systems, findings from older papers may not fully reflect current FBO engagement in these areas. Furthermore, the review represents literature from predominantly African countries, where the faith sector is well-integrated in national health systems [5]. While generalizability is limited to this geographical context, lessons drawn may be helpful in contexts with more fragmented faith sectors seeking to strengthen their health system engagement.

In addition, while the review quantified the extent of FBO engagement by counting the number of papers addressing specific pharmaceutical functions across different levels of the pharmaceutical system, it is critical to note that what is documented in the literature may not accurately reflect how roles are enacted in practice. Future research could explore how different types of FBOs across various levels of the pharmaceutical system differ in their contributions.

Finally, while the application of the PSS framework was valuable to provide a conceptual frame for the study, this work was also limited by the utility of the framework [10]. For example, the PSS framework does not distinguish between financing for pharmaceutical products and financing of broader pharmaceutical system operations – which is likely reflective of the pharmaceutical system’s integrated nature [9,10]. Nonetheless, distinguishing these aspects might help refine the understanding of FBOs’ contributions to pharmaceutical system financing and would be an interesting area for future exploration, particularly given the ongoing shifts in donor funding. For instance, future research might focus on an evaluation of the impact and scalability of drug revolving funds, one of the main income-generating models within FBOs.

Despite these limitations, this review is an essential step in mapping the scope of FBO engagement in pharmaceutical systems. Applying a comprehensive framework highlights key areas of contributions, the nature of contributions, gaps in representation, and areas for further inquiry, providing a foundation for further research. To our knowledge, this is the first application of the PSS framework, which was originally designed to assess national pharmaceutical systems, to the FBO context. This demonstrates the potential for adapting pharmaceutical system frameworks to the faith-based landscape, while underscoring the need for more research to facilitate appropriate contextualization.

Critically, the assessment highlights that across pharmaceutical components, FBOs frequently operate through collaborations and partnerships, highlighting the importance of prioritizing and enhancing strategic partnerships both within the faith sector and across sectors. This carries policy implications on two fronts. Faith actors should advance their advocacy for inclusion in programming, supported by rigorous measurement that produces quality data. Governments and regulators should create an enabling environment for such partnerships through, for example, memoranda of understanding with capable FBOs, in line with the Sustainable Development Goal 17 [62].

## Conclusion

This scoping review highlights the diverse roles of FBOs in pharmaceutical systems. Their most substantive contributions, as documented in the literature, lie in the PSS components: financing, regulatory, human resource, and provision of pharmaceutical products and related services. These contributions can be closely linked to the trust placed in FBOs within communities, underscoring the need for more research to understand trust dynamics, and for stronger partnerships between faith and health actors, as exemplified by the Georgetown-Lancet Commission on Faith, Trust and Health. Christian FBOs were overrepresented in the literature, demonstrating the need to have focused assessments on different religious groups that identify areas of alignment, divergence and opportunities for partnerships. Furthermore, a critical gap identified in this review is the lack of rigorous measurement of the outcomes and impact of FBO efforts, highlighting the need for mixed-methods studies that generate robust evidence to support governments in identifying, understanding and governing FBOs for stronger pharmaceutical systems. Therefore, this review provides a foundational mapping of FBO contributions, offering a critical starting point for future research and policy discussions on their role in pharmaceutical systems.

## Supporting information

S1 Checklist PRISMA checklist [16]. (DOCX)

S1 Table The Arksey and O’Malley approach [63]. (DOCX)

S2 Table Scoping Review Search Terms. (DOCX)

S3 Table Numerical summary analysis. (DOCX)

S1 Text Thematic Analysis Quotes. (DOCX)
