# Supplementary material for: The role of faith-based organizations in the pharmaceutical systems of low-and-middle-income countries – A scoping review
Source: PLOS Glob Public Health. 2026 Jul 27;6(7):e0006835. doi: 10.1371/journal.pgph.0006835 (PMC13405117; doi:10.1371/journal.pgph.0006835)
Supplement: S1 Table — (DOCX) [file pgph.0006835.s002.docx]

# S1_Table: The Arksey and O’Malley approach

| **Stage** | **Description** | **Application in this study** |
| --- | --- | --- |
| **Stage 1: Identifying the research question** | Specify all relevant aspects of the question to ensure that it is clear enough to guide the search strategy while also broad to provide comprehensive coverage in alignment with scoping review goals. | We defined the research question during the refinement of the dissertation topic, with guidance and input from the second and last authors of the paper. |
| **Stage 2: Identifying relevant studies** | Decide where to search, search terms to apply, time period and language. Sources can include electronic databases, organizations, conferences, targeted searching on key journals and references lists. | We refined search terms for select databases with the support of a Boston University Librarian. Additional documents were selected from relevant organizations, reference lists, and targeted searches. |
| **Stage 3: Study selection** | Apply post hoc inclusion and exclusion criteria as guided by the research question, and additional familiarity with the topic through reading studies. At least two reviewers should be involved at this stage. | Two reviewers independently reviewed the title and abstract of selected studies for relevance. Rayyan software was used to organize the screening process. |
| **Stage 4: Charting the data** | Develop a data charting form and use it to extract data from each study. Apply a ‘narrative review’ or ‘descriptive analytical’ method to extract contextual data from each study. | The PSS Framework was used as the basis of the data charting form. A numerical analysis describing the characteristics of included papers was undertaken. Thematic analysis was used to identify FBO contributions as described in the literature. |
| **Stage 5: Collating, summarizing, and reporting results** | Construct an analytic framework to provide an overview of the breadth of the studies. Present a numerical analysis characterizing the studies included, ensuring clarity and consistency. |  |
| **Stage 6: Consultation (optional)** | Engage stakeholders to suggest additional references and provide insights beyond those in the literature. | Two co-authors on this study are FBO experts who shared their insights, which were integrated into the results and discussion. |

Adapted from: Levac D, Colquhoun H, O’Brien KK. Scoping studies: advancing the methodology. Implement Sci. 2010;5:69. doi:10.1186/1748-5908-5-69
