# Supplementary material for: The role of faith-based organizations in the pharmaceutical systems of low-and-middle-income countries – A scoping review
Source: PLOS Glob Public Health. 2026 Jul 27;6(7):e0006835. doi: 10.1371/journal.pgph.0006835 (PMC13405117; doi:10.1371/journal.pgph.0006835)
Supplement: S2 Table — (DOCX) [file pgph.0006835.s003.docx]

# S2_Table: Scoping Review Search Terms

The scoping review search terms were applied from the year 2000 and beyond. The year 2000 was deemed appropriate as this marked the recognition of faith-based pharmaceutical service provision as a priority, with the establishment of the Ecumenical Pharmaceutical Network (EPN), a regional faith-based non-profit seeking to advance compassionate and just pharmaceutical services across low-and middle-income countries (63)

*Ecumenical Pharmaceutical Network [Internet]. Nairobi (KE): Ecumenical Pharmaceutical Network; c2018-2026 [cited 2024 May 6]. About us; [about 6 screens]. Available from:* [*https://www.epnetwork.org/about-us/*](https://www.epnetwork.org/about-us/)

|  | **Pharmaceutical functions** | **Faith-based organizations AND** | **Low- and middle-income countries** |
| --- | --- | --- | --- |
| **PubMed** | ("Pharmaceutical Services"[Mesh] OR Services, Pharmaceutic OR Services, Pharmacy OR Pharmaceutic Services OR Pharmaceutic Service OR Service, Pharmaceutic OR Services, Pharmaceutical OR Pharmaceutical Service OR Service, Pharmaceutical OR Pharmacy Services OR Pharmacy Service OR Service, Pharmacy OR Pharmaceutical Care OR Care, Pharmaceutical) OR ("Economics, Pharmaceutical"[Mesh] OR Pharmaceutical Economics OR Pharmacoeconomics OR Pharmacy Economics OR Economic, Pharmacy OR Economics, Pharmacy OR Pharmacy Economic) | ("Religion"[Mesh] OR Religions OR Religious Beliefs OR Beliefs, Religious OR Religious Belief OR Religious Ethics OR Ethic, Religious OR Prayer OR Prayers OR "Clergy"[Mesh] OR Ministers OR Minister OR Pastors OR Pastor OR Rabbis OR Rabbi OR Deacons OR Deacon OR Imams OR Imam OR Priests OR Priest OR Clerics OR Cleric OR Chaplains OR Chaplain OR "Religious Missions"[Mesh] OR Mission, Religious OR Missions, Religious OR Religious Mission OR Missions OR "Medical Missions"[Mesh] OR Medical Mission OR Mission, Medical OR Missions, Medical OR Medical Missions, Official OR Missions, Official Medical OR Official Medical Missions OR Medical Mission, Official OR Mission, Official Medical OR Official Medical Mission OR "Faith-Based Organizations"[Mesh] OR "Faith Based Organizations" OR "Faith-Based Organization" OR "Organization, Faith-Based" OR "Organizations, Faith-Based" OR "Faith Based" OR "Faith-Based") | afghan[Text Word] OR afghans[Text Word] OR afghani[Text Word] OR albanian[Text Word] OR albanians[Text Word] OR algerian[Text Word] OR algerians[Text Word] OR american samoan[Text Word] OR american samoans[Text Word] OR angolan[Text Word] OR angolans[Text Word] OR argentine[Text Word] OR argentines[Text Word] OR argentinian[Text Word] OR argentinians[Text Word] OR argentinean[Text Word] OR argentineans[Text Word] OR armenian[Text Word] OR armenians[Text Word] OR aruban[Text Word] OR arubans[Text Word] OR azerbaijani[Text Word] OR azerbaijanis[Text Word] OR bangladeshi[Text Word] OR bangladeshis[Text Word] OR bangalees[Text Word] OR belarusian[Text Word] OR belarusians[Text Word] OR byelorussian[Text Word] OR byelorussians[Text Word] OR belizean[Text Word] OR belizeans[Text Word] OR beninese[Text Word] OR benineses[Text Word] OR bhutanese[Text Word] OR bolivian[Text Word] OR bolivians[Text Word] OR bosnian[Text Word] OR bosnians[Text Word] OR botswana[Text Word] OR batswana[Text Word] OR brazilian[Text Word] OR brazilians[Text Word] OR brasilian[Text Word] OR brasilians[Text Word] OR bulgarian[Text Word] OR bulgarians[Text Word] OR burkinabe[Text Word] OR burkinese[Text Word] OR burundian[Text Word] OR burundians[Text Word] OR cape verdean[Text Word] OR cape verdeans[Text Word] OR cabo verdean[Text Word] OR cabo verdeans[Text Word] OR cambodian[Text Word] OR cambodians[Text Word] OR khmer[Text Word] OR cameroonian[Text Word] OR cameroonians[Text Word] OR central african[Text Word] OR central africans[Text Word] OR chadian[Text Word] OR chadians[Text Word] OR chinese[Text Word] OR colombian[Text Word] OR colombians[Text Word] OR comorian[Text Word] OR comorians[Text Word] OR congolese[Text Word] OR costa rican[Text Word] OR costa ricans[Text Word] OR ivorian[Text Word] OR ivorians[Text Word] OR cuban[Text Word] OR cubans[Text Word] OR djiboutian[Text Word] OR djiboutians[Text Word] OR dominican[Text Word] OR dominicans[Text Word] OR ecuadorian[Text Word] OR ecuadorians[Text Word] OR egyptian[Text Word] OR egyptians[Text Word] OR salvadoran[Text Word] OR salvadorans[Text Word] OR equatorial guinean[Text Word] OR equatorial guineans[Text Word] OR equatoguinean[Text Word] OR equatoguineans[Text Word] OR eritrean[Text Word] OR eritreans[Text Word] OR estonian[Text Word] OR estonians[Text Word] OR swazi[Text Word] OR swazis[Text Word] OR swati[Text Word] OR swatis[Text Word] OR ethiopian[Text Word] OR ethiopians[Text Word] OR fijian[Text Word] OR fijians[Text Word] OR gabonese[Text Word] OR gabonaise[Text Word] OR gambian[Text Word] OR gambians[Text Word] OR georgian[Text Word] OR georgians[Text Word] OR ghanaian[Text Word] OR ghanaians[Text Word] OR greek[Text Word] OR greeks[Text Word] OR grenadian[Text Word] OR grenadians[Text Word] OR guamanian[Text Word] OR guamanians[Text Word] OR guatemalan[Text Word] OR guatemalans[Text Word] OR guinean[Text Word] OR guineans[Text Word] OR bissau guinean[Text Word] OR bissau guineans[Text Word] OR guyanese[Text Word] OR haitian[Text Word] OR haitians[Text Word] OR honduran[Text Word] OR hondurans[Text Word] OR indian[Text Word] OR indians[Text Word] OR indonesian[Text Word] OR indonesians[Text Word] OR iranian[Text Word] OR iranians[Text Word] OR iraqian[Text Word] OR iraqians[Text Word] OR iraqi[Text Word] OR iraqis[Text Word] OR jamaican[Text Word] OR jamaicans[Text Word] OR jordanian[Text Word] OR jordanians[Text Word] OR kazakhstani[Text Word] OR kazakhstanis[Text Word] OR kenyan[Text Word] OR kenyans[Text Word] OR kiribati[Text Word] OR kiribatian[Text Word] OR kiribatians[Text Word] OR korean[Text Word] OR koreans[Text Word] OR kosovar[Text Word] OR kosovars[Text Word] OR kosovan[Text Word] OR kosovans[Text Word] OR kyrgyzstani[Text Word] OR kyrgyzstanis[Text Word] OR kyrgyz[Text Word] OR lao[Text Word] OR laotian[Text Word] OR laotians[Text Word] OR lebanese[Text Word] OR lesothan[Text Word] OR lesothans[Text Word] OR lesothonian[Text Word] OR lesothonians[Text Word] OR mosotho[Text Word] OR basotho[Text Word] OR liberian[Text Word] OR liberians[Text Word] OR libyan[Text Word] OR libyans[Text Word] OR macanese[Text Word] OR macedonian[Text Word] OR macedonians[Text Word] OR malagasy[Text Word] OR madagascan[Text Word] OR madagascans[Text Word] OR malawian[Text Word] OR malawians[Text Word] OR malaysian[Text Word] OR malaysians[Text Word] OR maldivian[Text Word] OR maldivians[Text Word] OR malian[Text Word] OR malians[Text Word] OR marshallese[Text Word] OR marshalleses[Text Word] OR mauritanian[Text Word] OR mauritanians[Text Word] OR mauritian[Text Word] OR mauritians[Text Word] OR mexican[Text Word] OR mexicans[Text Word] OR micronesian[Text Word] OR micronesians[Text Word] OR moldovan[Text Word] OR moldovans[Text Word] OR mongolian[Text Word] OR mongolians[Text Word] OR mongol[Text Word] OR montenegrin[Text Word] OR montenegrins[Text Word] OR moroccan[Text Word] OR moroccans[Text Word] OR mozambican[Text Word] OR mozambicans[Text Word] OR burmese[Text Word] OR myanma[Text Word] OR namibian[Text Word] OR namibians[Text Word] OR nepali[Text Word] OR nepalese[Text Word] OR nicaraguan[Text Word] OR nicaraguans[Text Word] OR nigerien[Text Word] OR nigeriens[Text Word] OR nigerian[Text Word] OR nigerians[Text Word] OR northern mariana islander[Text Word] OR northern mariana islanders[Text Word] OR mariana[Text Word] OR marianas[Text Word] OR pakistani[Text Word] OR pakistanis[Text Word] OR palauan[Text Word] OR palauans[Text Word] OR panamanian[Text Word] OR panamanians[Text Word] OR papua new guinean[Text Word] OR papua new guineans[Text Word] OR paraguayan[Text Word] OR paraguayans[Text Word] OR peruvian[Text Word] OR peruvians[Text Word] OR philippine[Text Word] OR philippines[Text Word] OR philipine[Text Word] OR philipines[Text Word] OR phillipine[Text Word] OR phillipines[Text Word] OR phillippine[Text Word] OR phillippines[Text Word] OR filipino[Text Word] OR filipinos[Text Word] OR filipina[Text Word] OR filipinas[Text Word] OR puerto rican[Text Word] OR puerto ricans[Text Word] OR russian[Text Word] OR russians[Text Word] OR soviet people[Text Word] OR soviet population[Text Word] OR rwandan[Text Word] OR rwandans[Text Word] OR rwandese[Text Word] OR ruandan[Text Word] OR ruandans[Text Word] OR ruandese[Text Word] OR samoan[Text Word] OR samoans[Text Word] OR sao tomean[Text Word] OR sao tomeans[Text Word] OR santomean[Text Word] OR santomeans[Text Word] OR senegalese[Text Word] OR serbian[Text Word] OR serbians[Text Word] OR montenegrin[Text Word] OR montenegrins[Text Word] OR sierra leonean[Text Word] OR sierra leoneans[Text Word] OR solomon islander[Text Word] OR solomon islanders[Text Word] OR somali[Text Word] OR somalis[Text Word] OR south african[Text Word] OR south africans[Text Word] OR south sudanese[Text Word] OR sri lankan[Text Word] OR sri lankans[Text Word] OR ceylonese[Text Word] OR saint lucian[Text Word] OR saint lucians[Text Word] OR vincentian[Text Word] OR vincentians[Text Word] OR sudanese[Text Word] OR surinamese[Text Word] OR surinameses[Text Word] OR syrian[Text Word] OR syrians[Text Word] OR tajik[Text Word] OR tajiks[Text Word] OR tajikistani[Text Word] OR tajikistanis[Text Word] OR tanzanian[Text Word] OR tanzanians[Text Word] OR tanganyikan[Text Word] OR tanganyikans[Text Word] OR thai[Text Word] OR timorese[Text Word] OR timoreses[Text Word] OR togolese[Text Word] OR tongan[Text Word] OR tongans[Text Word] OR tunisian[Text Word] OR tunisians[Text Word] OR turk[Text Word] OR turks[Text Word] OR turkish[Text Word] OR turkmen[Text Word] OR turkmens[Text Word] OR tuvaluan[Text Word] OR tuvaluans[Text Word] OR ugandan[Text Word] OR ugandans[Text Word] OR ukrainian[Text Word] OR ukrainians[Text Word] OR uzbek[Text Word] OR uzbeks[Text Word] OR vanuatu[Text Word] OR vanuatuan[Text Word] OR vanuatuans[Text Word] OR venezuelan[Text Word] OR venezuelans[Text Word] OR vietnamese[Text Word] OR yemeni[Text Word] OR yemenis[Text Word] OR yemenite[Text Word] OR yemenites[Text Word] OR yemenese[Text Word] OR yugoslav[Text Word] OR yugoslavs[Text Word] OR yugoslavian[Text Word] OR yugoslavians[Text Word] OR zambian[Text Word] OR zambians[Text Word] OR zimbabwean[Text Word] OR zimbabweans[Text Word] |
| **Web of Science: TS=(("Pharmaceutical Service*" OR "Pharmaceutic*" OR "Pharmacy Service*" OR "Pharmaceutical Care" OR "Pharmaco-economic*" OR "Pharmacy Economic*")**  **AND ("Religion*" OR "Spiritual*" OR "Faith*" OR "Belief*" OR "Prayer*" OR "Clergy*" OR "Minister*" OR "Pastor*" OR "Rabbi*" OR "Deacon*" OR "Imam*" OR "Priest*" OR "Chaplain*" OR "Mission*" OR "Faith-Based Organization*")**  **AND ("Developing Countr*" OR "Low-Income Countr*" OR "Middle-Income Countr*"))** | (TS=("Pharmaceutical Services" OR "Services, Pharmaceutic" OR "Services, Pharmacy" OR "Pharmaceutic Services" OR "Pharmaceutic Service" OR "Service, Pharmaceutic" OR "Services, Pharmaceutical" OR "Pharmaceutical Service" OR "Service, Pharmaceutical" OR "Pharmacy Services" OR "Pharmacy Service" OR "Service, Pharmacy" OR "Pharmaceutical Care" OR "Care, Pharmaceutical" OR "Economics, Pharmaceutical" OR "Pharmaceutical Economics" OR "Pharmacoeconomics" OR "Pharmacy Economics" OR "Economic, Pharmacy" OR "Economics, Pharmacy" OR "Pharmacy Economic") | TS=("Religion" OR "Religions" OR "Religious Beliefs" OR "Beliefs, Religious" OR "Religious Belief" OR "Religious Ethics" OR "Ethic, Religious" OR "Prayer" OR "Prayers" OR "Clergy" OR "Ministers" OR "Minister" OR "Pastors" OR "Pastor" OR "Rabbis" OR "Rabbi" OR "Deacons" OR "Deacon" OR "Imams" OR "Imam" OR "Priests" OR "Priest" OR "Clerics" OR "Cleric" OR "Chaplains" OR "Chaplain" OR "Religious Missions" OR "Mission, Religious" OR "Missions, Religious" OR "Religious Mission" OR "Missions" OR "Medical Missions" OR "Medical Mission" OR "Mission, Medical" OR "Missions, Medical" OR "Medical Missions, Official" OR "Missions, Official Medical" OR "Official Medical Missions" OR "Medical Mission, Official" OR "Mission, Official Medical" OR "Official Medical Mission" OR "Faith-Based Organizations" OR "Faith Based Organizations" OR "Faith-Based Organization" OR "Organization, Faith-Based" OR "Organizations, Faith-Based" OR "Faith Based" OR "Faith-Based")) | (TS=("developing countries" OR "developing countries")) |
| **Google Scholar** | “pharmaceutical services” | “faith based organizations” | “developing countries” |
| **Academic OneFile** | “pharmaceuticals and medical supplies” | "faith based organizations "OR Keyword: "faith based organisations" | "low and middle income countries" |
