# Supplementary material for: The role of faith-based organizations in the pharmaceutical systems of low-and-middle-income countries – A scoping review
Source: PLOS Glob Public Health. 2026 Jul 27;6(7):e0006835. doi: 10.1371/journal.pgph.0006835 (PMC13405117; doi:10.1371/journal.pgph.0006835)
Supplement: S3 Table — (DOCX) [file pgph.0006835.s004.docx]

# S3_Table: Numerical summary analysis

| **Paper Title** | **Author** | **Year** | **Study Design/Format** | **Country** | **Religious Group** | **Funding Source** |
| --- | --- | --- | --- | --- | --- | --- |
| Multi-country study of medicine supply and distribution activities of faith-based organizations in sub-Saharan African countries.(6) | WHO, EPN | 2006 | Cross-sectional study | Cameroon, DRC, Ghana, Kenya, Malawi, Nigeria, Rwanda, South Africa, Tanzania, Uganda, Zambia | Christianity | Public |
| Faith-based pharmaceutical supply chains and their role in African pharmaceutical systems: a qualitative systematic review.(7) | Jalloh I. et al. | 2024 | Systematic Review | Global | Christianity | Not reported |
| Pharmaceutical service delivery in church health systems in Africa: a cross-country analysis.(8) | Budge-Reid et al. | 2012 | Cross-sectional study | Ethiopia, Ghana, Kenya, Malawi, Nigeria, Tanzania, Togo, Uganda | Christianity | Public |
| Surveillance for falsified and substandard medicines in Africa and Asia by local organizations using the low-cost GPHF Minilab. (19) | Petersen A. et al. | 2017 | Cross-sectional study | Cameroon, Democratic Republic of Congo (DRC), Ghana, Kenya, Nigeria, Uganda, India | Christianity | Faith-based |
| Establishment of clinical pharmacy services: evidence-based information from stakeholders.(20) | Kilonzi et al. | 2024 | Commentary | Tanzania | Christianity | Non-governmental |
| Competitive strategies applied by Mission for Essential Drugs and Supplies (MEDS).(21) | Mwenda R. J | 2007 | Cross-sectional study | Kenya | Christianity | Not reported |
| An assessment of the implementation of the East African pooled procurement mechanism among faith-based medicines supply organizations.(22) | Jaguga C | 2018 | Cross-sectional study | Kenya, Rwanda, Tanzania, Uganda | Christianity | Not reported |
| Capacity building in health systems.(23) | Ecumenical Pharmaceutical Network | 2020 | Case study | Liberia, Rwanda, Nigeria, Kenya, DRC, Uganda, Zambia, | Christianity | Faith-based |
| Family planning and reproductive health supply stockouts: problems and remedies for faith-based health facilities in Africa.(24) | Metzger A.M. et al. | 2017 | Cross-sectional study | Cameroon, Central African Republic, Chad, DRC, Ethiopia, Kenya, Malawi, Niger, Nigeria, Tanzania, Uganda, Zambia, Zimbabwe | Christianity | Not reported |
| Building the evidence: unique stockout challenges of FBO health facilities.(25) | Christian Connections for International Health | 2015 | Cross-sectional study | Cameroon, Central African Republic, Chad, DRC, Ethiopia, Kenya, Malawi, Niger, Nigeria, Tanzania, Uganda, Zambia, Zimbabwe | Christianity | Multi-sector |
| Pooled procurement programme: Efficiency and challenges in medicinal health care-perspectives from National Catholic Health Service in Ghana.(26) | Domfeh et al. | 2021 | Case Study | Ghana | Christianity | Not sponsored |
| Pooled procurement program in the quality improvement of medicines of the National Catholic Health Service in Ghana: using the Donabedian model.(27) | Domfeh K et al. | 2021 | Case Study | Ghana | Christianity | Not reported |
| Multidisciplinary and multisectoral coalitions as catalysts for action against antimicrobial resistance: implementation experiences at national and regional levels.(28) | Joshi M.P. et al. | 2018 | Case study | Global | Christianity | Public |
| Promoting patient safety: medication without harm.(29) | Ecumenical Pharmaceutical Network | 2018 | Case study | Global | Christianity | Faith-based |
| Surveillance for substandard and falsified medicines by local faith-based organizations in 13 low- and middle-income countries using the GPHF Minilab.(30) | Gnegel G et al. | 2022 | Cross-sectional study | Kenya, Rwanda, Tanzania, Uganda, Burundi, Cameroon, Central African Republic, DRC, Chad, Ghana, Nigeria, Malawi, India | Christianity | Public |
| Low prevalence of substandard and falsified antimalarial and antibiotic medicines in public and faith-based health facilities of southern Malawi. (31) | Khuluza F et al. | 2017 | Cross-sectional study | Malawi | Christianity | Multi-sector |
| Identification of falsified chloroquine tablets in Africa at the time of the COVID-19 pandemic.(32) | Gnegel G. | 2020 | Commentary | Cameroon, DRC | Christianity | Public |
| Quality of essential medicines from different sources in Enugu and Anambra, Nigeria.(33) | Gabel J. et al. | 2024 | Cross-sectional study | Nigeria | Christianity | Multi-sector |
| Achieving high uptake of human papillomavirus vaccine in Cameroon: lessons learned in overcoming challenges.(34) | Ogembo J. et al. | 2014 | Case study | Cameroon | Christianity | Industry |
| “The refer less resolve more” initiative: A five-year experience from CMC Vellore, India.(35) | Velavan J | 2012 | Commentary | India | Christianity | Not sponsored |
| Health care providers’ attitude and satisfaction toward patient-oriented services provided by pharmacy technicians at three faith-based hospitals.(36) | Ntani S et al. | 2022 | Cross-sectional study | Cameroon | Christianity | Not sponsored |
| Identification and prioritization of critical success factors in faith-based and non-faith-based organizations’ humanitarian supply chain.(37) | Azmat et al. | 2019 | Thematic literature review, Cross-sectional study | Global | Islam, Christianity | Not reported |
| Prescribing habits in church-owned primary health care facilities in Dar Es Salaam and other Tanzanian Coast regions.(38) | Massele AY et al. | 2001 | Cross-sectional study | Tanzania | Christianity | Non-governmental |
| Faith-based organizations as partners in family planning: working together to improve family well-being.(39) | Institute for Reproductive Health | 2017 | Cross-sectional study | Global | Christianity, Islam | Public |
| Awareness, acceptability and uptake of human papilloma virus vaccine among Cameroonian school-attending female adolescents.(40) | Ayissi et al. | 2012 | Cross-sectional study | Cameroon | Christianity | Industry |
| Engaging faith-based organizations for promoting the uptake of COVID-19 vaccine in India: a case study of a multi-faith society.(41) | Soni G. et al. | 2023 | Case Study | India | Islam, Sikhism, Christianity, Hinduism, Buddhism | Multi-sector |
| Religion is not a barrier to family planning.(42) | Joshi S. | 2017 | Commentary | Global | Christianity, Islam | Not reported |
| The role of faith-based organizations in improving vaccination confidence & addressing vaccination disparities to help improve vaccine uptake: a systematic review.(43) | Syed U et al. | 2023 | Systematic Review | Ethiopia, Ghana, Indonesia, South East Asia | Christianity | Not sponsored |
| Family planning methods and fertility preferences according to HIV status among women in Cameroon.(44) | Ruark A. et al. | 2021 | Cross-sectional study | Cameroon | Christianity | Faith-based |
| Availability of family planning services and quality of counseling by faith-based organizations: a three country comparative analysis.(45) | Barden-O'Fallon J. | 2017 | Cross-sectional study | Malawi, Kenya, Haiti | Christianity | Public |
