# Supplementary material for: The role of faith-based organizations in the pharmaceutical systems of low-and-middle-income countries – A scoping review
Source: PLOS Glob Public Health. 2026 Jul 27;6(7):e0006835. doi: 10.1371/journal.pgph.0006835 (PMC13405117; doi:10.1371/journal.pgph.0006835)
Supplement: S1 Text — (DOCX) [file pgph.0006835.s005.docx]

# S1_Text: Thematic Analysis Quotes

## Component 1: Financing

Donations:

*“Of the FB-DSOs outlined in Table 4 and Figure 3 (N=12), nine were supported entirely through donor funds, while five also received national government funding, and a further nine used sales of medicines as an additional revenue stream”* – Jalloh et al., 2024

*“Half the DSOs received drug donations for distribution to their customers free of charge, and the study revealed that these donations could create problems rather than responding to real needs”* – WHO, EPN, 2006

Drug revolving funds:

*“The drug revolving fund was established in 1986 by donor partners who included World Health Organization (WHO), World Bank (WB) and Danish International Aid Agency (DANIDA). This constitutes the capital fund for the organization. The fund has grown over the years from Kshs 200 million in 1986, through retained surplus to match the current level of operations (to Kshs.750 million in 2006).”* – Mwenda et al., 2007

Diversifying funding sources:

*“Additionally, following the initial 2008 kick-off meeting, EPN worked to sustain their AMR[antimicrobial resistance]-related efforts by successfully diversifying their funding with support from multiple partners (Action on Antibiotic Resistance, Bread for the World, Difaem, Misereor, SPS, and SIAPS).”* – Joshi et al., 2018

Pooled procurement:

*“I can say that PPP [Pooled Procurement Programs], by and large, is a very good innovation or intervention and it has come to stay and over the years facilities have been able to achieve at least 30% in the cost of reduction of medicine and medical consumables and this is very appreciable success and a lot more can be achieved if facilities patronize the program holistically and government for that matter can improve upon payment.”* – Domfeh, 2021

*“The major challenges were conflicting legislations and regulations for health products in East African countries and limited financial resources for procuring health products. The moderate challenges were quantification and forecasting; pre-qualification of suppliers; tendering approach and adjudication; price negotiations; monitoring order status; receiving order status; receiving products and reporting, monitoring and evaluation. The only minor challenge was harmonization of products.”* – Jaguga, 2018

## Component 2: Regulatory systems

Quality and safety surveillance:

*“Sixteen samples showed non-compliance in disintegration testing. In two of these cases, the tablets had not disintegrated even after two days. The faith-based drug supply organization decided to contact the local manufacturer, presented the test result, and the manufacturer thereupon issued a product recall…”* – Gnegel et al., 2022

*“In March 2020, local member organizations of the EPN [Ecumenical Pharmaceutical Network] reported that both in private pharmacies and in informal markets, several types of falsified CQ [chloroquine] tablets were appearing which, in local GPHF Minilab analysis, 11 were found not to contain CQ. Through the German Institute for Medical Mission (Difaem), the member organization of EPN which coordinates the Minilab network, the WHO [World Health Organization] Rapid Alert System, was informed, and the WHO published an international Medical Product Alert about falsified CQ tablets.”* – Gnegel et al., 2020

*“In the present study, the single falsified medicine sample, which was identified was collected in the informal sector, that is, from a non licensed street vendor. In addition, five substandard medicines were found in different parts of the private sector (two licensed pharmacies, two licensed drug stores, and one non licensed street vendor…Our finding of a low prevalence of substandard and falsified medicines in public and CHAM [Christian Health Association of Malawi] health facilities may help to restore the trust of both the population and the health workers in the medicines provided in these facilities.” ­*– ­­Khuluza et al., 2017

*“Since 1998 the Global Pharma Health Fund in Germany distributed about 800 Minilab kits to 97 different countries. They are used by governments, international institutions, NGOs and others. But according to GPHF, the DIFAEM EPN Minilab Network is the only group actively presenting the results. Dr. Richard Jaehnke, “father” of the Minilab and winner of the 2017 “Humanity in Sciences” award, states in an article “Taking down Goliath”: “I find with church groups, there is more of a rapport – […] and it´s more transparent. […] They track fake medicines down even more effectively than the police – because in their eyes, delivering counterfeit medicine with nothing inside is ´like cheating God.”* – Ecumenical Pharmaceutical Network, 2018

## Component 4: Pharmaceutical products and related services

Procurement and distribution:

*“Faith based organizations in three African countries (Malawi, Kenya and Uganda) were, in the late nineties, estimated to handle between 20 percent and 40 percent of their country’s flow of pharmaceuticals”* – Budge-Reid, 2012

Selection, procurement and distribution:

*“Among the major findings was that the proportion of the population served by 15 faith‐ based DSOs in 10 countries ranged from 25‐60%, with an average of 43%. This figure indicates that public medicines supply systems do not cover the entire population and that faith‐based supply organizations are a necessary complement to public systems.”* – WHO, EPN, 2006

Procurement:

*“Two joint bulk purchasing exercises were conducted in 2015 and 2016 respectively, but were characterized by minimal price reductions, inconsistent supply of health products and administrative challenges. These and other undocumented challenges had largely contributed to the East African pooled procurement mechanism’s (EAPPM) failure to achieve meaningful impact years after formation.”* – Jaguga, 2018

Distribution:

*“Non-religious organizations when dealing with religiously motivated beneficiaries come across challenges and hurdles that faith-based organizations could easily avoid especially on the local, regional and national levels, as they mostly consist of the trusted members of the society and beneficiaries tend to trust them more than secular organizations if they belong to the similar faith group.”* – Azmat et al., 2019

## Component 5: Information

Computerized systems:

*Of the 12 DSOs with drug inventory control systems, eight had computerized inventory control systems and four still did manual inventory control checks.* – WHO, EPN, 2006

*MEDS Head of Finance and ICT pointed out that the organization is 100% computerized. The ICT infrastructure has enhanced the speed and efficiency of communication with customers, donors and other stakeholders. ICT has also improved data and record keeping. Overall, it has increased the efficiency of operations and service delivery.* – Mwenda, 2007

Digital systems:

*“Analysis of the supportive supervision findings revealed that 5 of the 17 visited hospitals have created a system for pharmacists to access patient information through the hospital information management system (HIMS).”* – Kilonzi et al., 2024

Data collection, processing and dissemination:

*“Again, we have a problem with submission of data, currently the secretariat does not have a system whereby we can determine in real-time the demand and supply between facilities and suppliers and ability to track payment as and when they are due and as and when payment is effected, so we rely on facilities and suppliers alike to send data for the program to appraise itself of what is happening.Coordinator of Pooled Procurement Program, DSO”* – Domfeh, 2021

*“‘Another challenge is, the report that we are supposed to send because of our location it makes internet connection very difficult and reports to be sent to the NCHS are usually sent out of the hospital. Which is not very appropriate. They should help with the internet accessibility’ Supply Officer, Health Facility”* – Domfeh 2021

Data collection and processing:

*“Four of the Cameroonian FBOs have been trained on the country’s UNFPA forecasting system to enable them to strengthen and organize their internal systems to monitor and collect data necessary to participate in the national supply chain systems. This allows them to participate in the national process for forecasting and receiving commodities*.” – Metzger et al., 2017

## Component 6: Policy, laws and governance

Coordination and leadership:

*“EPN leadership promoted and coordinated activities within its network of faith-based pharmaceutical organisations…"EPN developed public messaging on AMR [antimicrobial resistance] and 10 comic strips as a vehicle for dissemination. Over 33,000 comic strips [raising awareness on antimicrobial resistance] were shared across EPN’s member network [FBOs at different levels of the pharmaceutical system and religious leaders] in 35 countries and in six languages…EPN’s pre-existing network of faith-based pharmaceutical organisations and professionals was a structure that naturally lent itself to including AMR as a valuable addition to its other priorities. EPN leadership then successfully brought about action by providing information, motivation, and technical support to its network of members. "* – Joshi et al., 2018

*“Seven Cameroonian faith-based organizations started meeting in early 2016 with an initial introduction to the SMART family planning advocacy framework from Advance Family Planning. They developed group goals and objectives to move forward as a group of Cameroonian FBOs to reduce stockouts in their facilities. They then formally organized themselves as the Alliance of Christian Faith-Based Organizations for Family Planning (ACFBOFP) and registered with the government. This group developed a constitution, internal rules, a governing body, and training on family planning methods for its members. It continues to meet on advocacy strategy and is recruiting additional FBOs to the alliance.”* – Metzger et al., 2017

Advocacy:

*“We organized an advocacy event with policy makers where we were overwhelmed by the response. We were given a small room and so many individuals attended that we could not all fit. Some conservative policy makers were even in attendance. A Catholic colleague told us that the way we presented family planning was the first time that he’s been in a meeting where family planning was presented in such a way that he thinks Catholics would be comfortable – Christian (Ecumenical) FBO, global”* – Institute for Reproductive Health, 2021

## Component 7: Innovation, research and development and manufacturing

Manufacturing:

*“Of the 16 DSOs, six reported that they had local production units to manufacture a range of products.”* – WHO, EPN, 2006

## Pharmaceutical System Attributes: Performance and Resilience

Performance:

*“When there is improvement in the payment of NHIS [National Health Insurance Scheme] claims, the PPP [pooled procurement program] will also improve because one of the reasons why we have not been able to drive the discounts further down is because of the delay in the payment to suppliers. – Procurement Manager, DSO”* – Domfeh et al., 2021

Resilience:

*“Disaster preparedness procedures in place: 27 percent have a plan for what to do in the event of a large-scale natural disaster, such as flooding, or conflict-based emergencies.”* – Budge-Reid et al., 2012

## Pharmaceutical System Outcomes: Access and Use

Access (Acceptability):

*“In the short run, the influence of religious leaders was utilized to garner support for COVID-19 vaccination and build confidence in vulnerable communities by highlighting the vaccine’s safety and efficacy…The immediate impact of engaging faith leaders and faith-based organizations was vaccinating 0.41 million beneficiaries of eligible cohorts till January 2023. This was done through awareness campaigns that sensitized many FBO leaders and their followers on the efficacy and safety of the vaccines.”* – Soni et al., 2023

*“In Egypt, the Al-Azhar mosque and Al-Azhar University in Cairo have regularly issued fatwas (Islamic decrees) to promote the use of modern contraception. Almost half of all Egyptian women now rely on modern contraception.”* – Joshi, 2017

*“In Rwanda, the Presbyterian Church organized family-planning workshops in collaboration with the Ministry of Finance. In a speech at the workshop in 2010, Pastor Emmanuel Muhozi, who was in charge of training, said: ‘The word of God doesn’t contradict the message of family planning. The Bible calls upon parents to be blessings to their children, not a curse.’ Driving home the point, he asked: “How will a parent with uncountable children ever bless them in any way?’“* – Joshi, 2017

Use:

*“The most significant long-term outcome has been the ownership of the vaccination program by faith-based leaders. This will ensure the sustained participation of the FBOs in the current and future public health crises and vaccination drives.”* – Soni et al., 2023

*“FBOs are able to interact with the community to deliver an evidenced-based intervention that has been modified for cultural relevance and to emphasize medication adherence…There should be sustained engagement and unique inputs of various communities, from small informal groups at the grassroots level right up to global coalitions. Indonesia’s two largest Islamic FBOs have strengths such as their organizational structure to target the grassroots… Importantly, the Central Board of one of the FBOs provided support for COVID-19 vaccination implementation.”* – Syed et al., 2023

Access and use:

*“The only sensitivity is about where does one begin - from what age can we make methods available? Churches generally are uncomfortable with offering methods to young, unmarried people. But once someone is pregnant, we generally agree that they should be given information and access to methods.”* – Institute of Reproductive Health, 2011

*“There are challenges. Because we would like a more aggressive approach in terms of providing information, especially to the youth. But churches might want a very slow and conscious approach. The churches might want to be in charge of the process through their own churches and systems, so that the information is given as they want it.”* – Institute of Reproductive Health, 2011

Access (Availability):

“*Also, smaller health facilities benefited from joining the association by conserving some valuable resources and channeling them to other areas of the health system. Therefore, through solidarity within the PPP mechanism, some health facilities with weaker financial muscles were able to acquire the medicines needed.”* – Domfeh, 2021

*“The availability of quality-assured medicines and medical supplies in those DRF (Drug Revolving Fund) health facilities has improved. A survey in 8 selected facilities observed an improvement of availability of essential medicines from 36% to 75% on average”* – Ecumenical Pharmaceutical Network, 2020

*“Church facilities had significantly higher scores than other groups for drug availability. Thus, for example, there was a 90% chance of their having chloroquine, and a 70% chance of having penicillin constantly available.”* – Jalloh et al., 2024

*“Twelve of the 16 RH (reproductive health) products were used by at least 80 percent (37) of the FBO facilities. These were male condoms, injectable contraceptives, combined and progestin-only oral contraceptives, contraceptive implants, erythromycin, clotrimazole, magnesium sulfate, methyldopa, misoprostol, nifedipine, and oxytocin”* – Metzger et al., 2017

*“The availability of FP services by faith-based facilities varied by country. For example, the percent of faith-based facilities offering FP services in Malawi was 57%, as compared to 95% public and 77% other private sector. In Kenya the percent of faith-based facilities offering FP services was 69%, as compared to 97% public and 83% other private sector. In Haiti, the percentage of faith-based facilities offering FP services was 89%, as compared to 93% in the public sector and 72% in other private facilities.”* – Barden-O’Fallon, 2017

*“However, many customers indicated that only 0 – 50% of the number of items and between 50 – 100% of the quantities of items ordered were met by DSOs*.” – WHO, EPN, 2006

## Additional pharmaceutical system themes: measurement and collaboration

Measurement:

*“Secular respondents were more likely to describe their organizational mandate as contributing to national maternal and child health, family planning and other reproductive health goals and strategies, whereas FBOs were more likely to see these issues first and foremost through the lens of their faith which tends to emphasize hard-to-measure aspects like the quality of relationships and improvement not only of families’ physical but also spiritual well-being.”* – Institute for Reproductive Health, 2011

Collaboration:

*“In 2021, MUHAS, in collaboration with the Christian Social Services Commission (CSSC) and with the support of action medeor e.V of Germany, implemented a project to strengthen the provision of CPS in public and faith- based hospitals in Tanzania.”* – Kilonzi et al., 2024

*“The MOHs [Ministries of Health] (compared to FBSOs [faith-based supply organizations], international sources, or other sources) were the most common suppliers of oral contraceptive pills and CycleBeads®. In fact, 50 percent or more of the facilities reported that their MOH supplied all their contraceptive products, with the exception of IUDs [intrauterine devices].”* – Metzger et al., 2017

*“The study showed that customers had multiple sources of supply besides DSO supply services and that they supplemented their stocks with government supplies or buying from private wholesalers.”* – WHO, EPN, 2006
